# Exercise as ‘precision medicine’ for insulin resistance and its progression to type 2 diabetes: a research review

**DOI:** 10.1186/s13102-018-0110-8

**Published:** 2018-11-23

**Authors:** Fred J. DiMenna, Avigdor D. Arad

**Affiliations:** 1 Division of Endocrinology, Diabetes and Bone Disease, Icahn School of Medicine at Mount Sinai, 1111 Amsterdam Avenue, Babcock 10th Floor, Suite 1020, New York, 10025 New York USA; 2 Department of Biobehavioral Sciences, Columbia University Teachers College, 525 W. 120th Street, New York, 10027 New York USA

**Keywords:** Type 2 diabetes, Insulin resistance, Obesity, Intramyocellular lipid, Mitochondrial dysfunction, Ectopic lipid accumulation, Metabolically-healthy obesity, Fatmax, Critical power/velocity, High-intensity interval training

## Abstract

Type 2 diabetes and obesity epidemics are in effect in the United States and the two pathologies are linked. In accordance with the growing appreciation that ‘exercise is medicine,’ it is intuitive to suggest that exercise can play an important role in the prevention and/or treatment of these conditions. However, if exercise is to truly be considered as a viable alternative to conventional healthcare prevention/treatment strategies involving pharmaceuticals, it must be prescribed with similar scrutiny. Indeed, it seems reasonable to posit that the recent initiative calling for ‘precision medicine’ in the US standard healthcare system should also be applied in the exercise setting. In this narrative review, we consider a number of explanations that have been forwarded regarding the pathological progression to type 2 diabetes both with and without the concurrent influence of overweight/obesity. Our goal is to provide insight regarding exercise strategies that might be useful as ‘precision medicine’ to prevent/treat this disease. Although the etiology of type 2 diabetes is complex and cause/consequence characteristics of associated dysfunctions have been debated, it is well established that impaired insulin action plays a critical early role. Consequently, an exercise strategy to prevent/treat this disease should be geared toward improving insulin sensitivity both from an acute and chronic standpoint. However, research suggests that a chronic improvement in insulin sensitivity only manifests when weight loss accompanies an exercise intervention. This has resonance because ectopic fat accumulation appears to represent a central component of disease progression regardless of whether obesity is also part of the equation. The cause/consequence characteristics of the relationship between insulin resistance, pathological fat deposition and/or mobilsation, elevated and/or poorly-distributed lipid within myocytes and an impaired capacity to use lipid as fuel remains to be clarified as does the role of muscle mitochondria in the metabolic decline. Until these issues are resolved, a multidimensional exercise strategy (e.g., aerobic exercise at a range of intensities and resistance training for muscular hypertrophy) could provide the best alternative for prevention/treatment.

## Introduction

Recent statistics indicate that 23.1 million people (7.2% of the population) in the US have diabetes with another 7.2 million afflicted, but not diagnosed [1]. The vast majority possess type 2 diabetes (T2D), a disorder that is linked with the ‘American obesity epidemic.’ Moreover, ~ 34% of American adults have prediabetes, which means they are at increased risk for developing T2D and comortalities including heart disease and stroke [1]. Indeed, estimates are that 25% of those diagnosed with prediabetes will have T2D within 3–5 years. The end result is that diabetes was responsible for ~ 12% of deaths in the US in 2010 making it the third leading cause of mortality in the country [2].

**Fig. 1:**
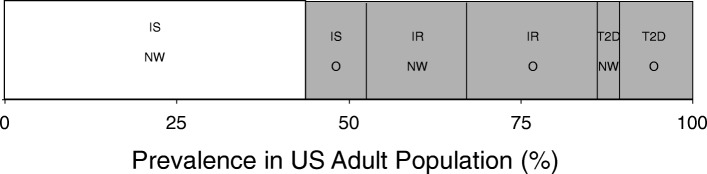
Prevalence of insulin resistance, obesity and type 2 diabetes in the US adult population. The complex interaction between insulin resistance, overweight/obesity and type 2 diabetes is exemplified by five distinct subsets of metabolically-compromised individuals that collectively comprise > 50% of the US adult population (grey shade). The fact that some of these individuals can fend off metabolic decline despite being obese while others possess type 2 diabetes and/or insulin resistance without obesity provides evidence that initiation and progression of type 2 diabetes is multi-factorial and complex. IS, insulin sensitive; IR, insulin resistant; T2D, type 2 diabetes; NW, normal weight; O, obese

With respect to treating both symptom and cause, the association between T2D and obesity is important to explore. Overweight/obesity is a well-established risk factor that accounts for 80–85% of the likelihood of developing T2D [3, 4]. However, while most individuals with T2D are obese, most obese individuals do not develop T2D despite demonstrating an impaired capacity for insulin-regulated gluco/lipometabolism in target organs/tissues (i.e., insulin resistance; IR) [5]. Furthermore, there is a growing body of research exploring atypical patients for whom the obesity/IR association is disjointed; for example, obese individuals with normal insulin sensitivity (IS) [6–8] and individuals with IR who are not overweight [9–11] (i.e., ‘metabolically-healthy obese’ and ‘metabolically-unhealthy normal weight,’ respectively). Finally, T2D has even been reported in non-obese subjects without IR due to deficiencies in insulin secretion [12, 13]. Collectively, these observations regarding distinct subsets of metabolically-compromised individuals (see Fig. 1) reflect the highly complex and multifactorial aetiology of T2D in obese and non-obese individuals.

In 2007, a health initiative was launched jointly by the American Medical Association (AMA) and American College of Sports Medicine (ACSM) [14]. Termed ‘Exercise is Medicine,’ the premise was that instead of a healthcare system based on ‘sick care,’ the focus should be on preservation and promotion of health with exercise playing a major role [14]. Needless to say, with the aforementioned epidemics in effect and this sentiment resonating throughout the field, a considerable amount of recent research has been directed toward defining the role that exercise can play in treating IR and delaying/preventing its progression to T2D. This appears appropriate because physical inactivity has been identified as one of six risk factors for complications of T2D for US adults [1]. However, while it is intuitive to believe that exercise can benefit the health profile of these patients, there is evidence to suggest that exercise per se might not be enough. For example, some studies have shown that chronic aerobic training does not improve IR unless hypocaloric intake and corresponding weight loss accompanies the intervention [15]. Moreover, IR can be improved by surgical removal of body fat [16, 17], which means that it is the alteration in fat deposition, as opposed to chronic negative energy balance per se, that is the stimulus that drives the associated improvement. Therefore, in the absence of weight loss, the degree to which chronic aerobic training and/or resistance training (RT) can influence the pattern of fat deposition both in adipose [18] and muscle [19] tissue is an important factor to consider.

The purpose of this article is to review research exploring the role that chronic exercise training can play in the prevention/treatment of IR and its progression to T2D. In doing so, we will pay particular attention to the link between obesity and T2D with emphasis devoted to the metabolically-healthy obese and metabolically-unhealthy normal-weight phenotypes that serve to dissociate the two pathophysiologies. We will also explore the strong genetic predisposition for IR in the offspring of individuals with T2D (FH+). An underlying theme will be addressing the ongoing debate regarding the role of muscle mitochondria in the pathological progression.

### Review of exercise as precision medicine for insulin resistance and its progression to type 2 diabetes

**Insulin-resistant Glucose Metabolism**

Generally speaking, T2D involves a combination of IR and inadequate insulin secretion to adequately compensate [20]. The progression to T2D has been suggested to comprise five definitive stages with IR initiating a pathological decline that requires years or even decades to complete [21]. The critical role of IR in this sequence is exemplified by the fact that it underpins the strong genetic predisposition for T2D [22] with FH+ demonstrating marked IR long before glucose tolerance becomes impaired [23, 24]. Indeed, in accordance with this genetic link, IR has been advanced as a significant contributor to the T2D progression [25]. In stage 1, IR begins when insulin-sensitive target tissues (e.g., skeletal muscle, liver and adipocytes) begin to demonstrate a decreased response to the regulatory prowess of the circulating hormone. Particularly relevant in this regard is skeletal muscle, which represents the predominant site of insulin-stimulated glucose disposal in the body. At this early stage of disease progression, the measurement of C-peptide (a byproduct of insulin production that exists in equal amount) concentration in blood or urine might provide a useful way to identify the need for a prophylactic exercise intervention. Fortunately, in this initial ‘compensation’ stage, increased insulin secretion (hyperinsulinemia) secondary to an increase in pancreatic β-cell mass/productivity allows for maintenance of homeostatic levels of plasma glucose. Left unaddressed, however, the chronic overload associated with this ‘quick fix’ takes its toll and β-cells become dysfunctional [26] with glucose-stimulated insulin secretion ultimately reduced (stage 2) [21]. The resultant hyperglycemia continues the pathological progression and allows for the diagnosis of prediabetes via measurement of fasting plasma glucose and/or hemoglobin A1c (i.e., tests that identify elevations in plasma-glucose concentration). With the passage of time, β-cell function continues to decline with increasing IR (stage 3) causing a cascade of events (stage 4) that ultimately culminates in T2D (stage 5) [21].

### Type 2 diabetes as a disease of lipid metabolism

With IR and resultant hyperglycemia playing a critical role in the pathological progression to T2D, it is not surprising that the disease is considered one of aberrant glucose metabolism. However, for prevention/treatment purposes, it might be short-sighted to define diabetes as a disease of carbohydrate metabolism alone. Indeed, there is growing support for an ‘alternative angle’ offered in 1992 when J. Denis McGarry asked the whimsical question, ‘What if Minkowski had been ageusic?’ [27]. In his opinion piece, McGarry speculated a series of events based on Randle’s substrate-competition model [28] whereby an increase in lipid oxidation would inhibit glucose use. Within this schema, IR-induced hyperinsulinemia and resultant liver lipogenesis and synthesis of very-low-density lipoproteins (VLDL) would serve to increase the flux of triglycerides into storage sites including muscle (intramyocellular lipid; IMCL) thereby interfering with normal glucose processing. The end result would be an elevation of plasma glucose that completes a vicious cycle of aberrant fuel partitioning [27].

McGarry’s contention that the pathophysiological mechanisms of IR and hyperglycemia should be considered offshoots of dysfunctional lipid metabolism is supported by a number of more recent observations. For example, using nuclear magnetic resonance spectroscopy, Krssak et al. confirmed an inverse relationship between IS and plasma free fatty acid concentration (FA) during fasting in normal-weight adults without T2D [29]. Importantly, an inverse relationship was also found between IS and IMCL in this study [29]. Similarly, Perseghin et al. used nuclear magnetic resonance spectroscopy to show that in lean insulin-resistant FH+ (i.e., a group at high risk for developing the disease [30]), the main predictors of whole-body IS were FA and intramyocellular triglyceride content (IMTG; i.e., the predominant component of IMCL) [31]. Interestingly, a similar association between IMTG and IR was reported independent of total-body adiposity in Pima Indian men without T2D, a group that is predisposed to obesity and development of the disease [32]. Finally, longitudinal studies involving dietary interventions that increase [33] or decrease [34] lipid storage confirm an inverse relationship between IMCL and IS. The reason for this link between elevated IMCL and IS is unclear, but likely reflects a ‘lipotoxic’ chain of events initiated by lipid metabolites (e.g., long-chain fatty acyl-CoA, ceramides, sphingosine-1-phosphate and some diacylglycerol species) which activate a serine-kinase cascade that desensitises insulin receptors [35–38].

### The paradox of IMTG accumulation

While it is attractive to implicate the lipotoxic effects of metabolites that accompany elevated IMCL as the precursor of IR, it is important to note that endurance-trained subjects who demonstrate high IS also have elevated IMCL [35, 39–41]. A likely explanation for this ‘athlete’s paradox’ is that both energy turnover and the capacity to satisfy energetic requirements via lipid oxidation are markedly greater for endurance-trained compared to untrained subjects. Consequently, it is the degree to which IMCL is used as opposed to storage per se that appears responsible for pathological repercussion when stores are elevated. In this regard, Stannard and Johnson advance a teleological model of IR based on the contention that it represents a functional adaptation that occurs in response to chronic energy surplus [42]. Their reasoning is based on the ‘thrifty-genotype hypothesis,’ which posits a hunter/gatherer phenotype for which periods of food abundance are inevitably followed by periods of food scarcity [42]. During the latter periods, elevations in IMCL and reductions in IS that develop during the former provide for an easily-accessed energy source and conservation of glucose, respectively [42, 43]. However, when the food-scarcity component is removed from the equation for the untrained individual (e.g., as is the case in the present day with food procurable without significant energetic outlay and hunting and gathering having been replaced by sitting and lying down), IMTG stores are not adequately used, glucose is preserved in abundance and the response to energetic surplus that had been functional becomes pathological. Endurance athletes avoid this consequence by maintaining a physically-active (and, therefore, physiologically ‘normal’) lifestyle. Given our increasingly sedentary existence and the associated increase in likelihood of hyperenergetic intake, evaluation of this athletic phenotype appears particularly relevant.

### Skeletal muscle mitochondrial dysfunction in patients with type 2 diabetes

While the association between family history and T2D is strong, how the majority of the inherited risk is conveyed remains to be determined [44]. Although environmental factors dictated by parental input (e.g, diet and physical activity) cannot be discounted, if IR is a significant contributor to the T2D progression and metabolites associated with elevated IMCL beget IR, the cause of pathological IMCL accumulation might be a significant factor responsible for passing the predisposition for T2D in some cases. In accordance with basic supply/demand dynamics, it is intuitive that excess IMCL accumulation could result from an overabundance of FA availability/uptake to/by muscle and/or insufficient FA oxidation within muscle. While the former could play a role [45, 46], most research interest has been devoted to the latter with evidence suggesting that patients with T2D demonstrate a reduced capacity for lipid oxidation during the fasting [47, 48], postprandial [47] and exercise [49] conditions. Consequently, as the organelles responsible for lipid oxidation, skeletal muscle mitochondria have been implicated as potential culprits. Specifically, the reduced capacity for lipid oxidation could result from ‘mitochondrial dysfunction;’ for example, decreased mitochondrial biogenesis, reduced mitochondrial content and/or decreased protein content/activity of oxidative enzymes per unit mitochondria [50]. Support for this contention comes from Simoneau and Kelley who used percutaneous biopsy of the vastus lateralis to confirm lower oxidative-enzyme activity in subjects with T2D compared to counterparts without the disease [51]. Moreover, the ratio of glycolytic-to-oxidative enzymes was negatively correlated with IS with the ratio of citrate synthase to hexokinase providing the strongest correlation [51]. Abnormal hexokinase activity has also been implicated in IR [52]. In a follow-up study, Kelley et al. also obtained muscle biopsies and found lower overall respiratory-chain activity and smaller intermyofibrillar mitochondria (i.e., the mitochondrial subpopulation that provides energy for muscle contractile activity [53]) in the group with T2D with each of these decrements positively correlated with the severity of IR [54]. In a similar study, Ritov et al. isolated mitochondrial subpopulations and found that respiratory-chain activity for patients with T2D was particularly compromised in the subsarcolemmal subpopulation, the fraction that provides energy for insulin-signaling processes like signal transduction, ion exchange and substrate transport/activation [55]. In this study, patients with T2D also demonstrated decreased subsarcolemmal mitochondrial content; however, importantly, this reduction did not fully explain the decrease in activity [55]. Finally, Mogensen et al. induced state-3 respiration with pyruvate plus malate in fully-coupled isolated mitochondria and maximal respiration through the electron transport chain to confirm a reduction in function per mitochondrion for patients with T2D compared to subjects that did not possess the disease [56]. Moreover, the values were associated with HbA1c measurement, which is consistent with a link between mitochondrial dysfunction and the chronic elevation of blood-glucose concentration that the HbA1c measurement identifies [56].

A T2D-related deficit attributable to a reduction in mitochondrial functional capacity, as opposed to content per se, is an important finding because it is consistent with the notion that mitochondria convey the inherent defect that predisposes T2D. However, there are a number of caveats when interpreting observations from these investigations. In all cases, the patients with T2D were also obese, which is important because obesity adversely affects mitochondrial function independent of T2D status. For example, in three of the four studies, three groups were included for comparison such that the effect of obesity without T2D could be isolated and in these studies, the decrements observed for the obese subjects with T2D were also present for the obese subjects without the disease although reductions in overall [52, 54] and subsarcolemmal [55] activity were greater when obesity was accompanied by the disease. It is also insightful to note that Oberbach et al. report a fiber-specific ratio of glycolytic-to-oxidative enzyme activity that was not different for patients with T2D compared to healthy control subjects matched for age- and body mass index (BMI) [57]. However, patients with T2D demonstrated a 16% reduction and 49% increase in slow-oxidative and fast-glycolytic fiber fraction, respectively [57]. Although the authors’ use of glycerol-3-phosphate dehydrogenase as a marker of glycolytic enzyme activity can be questioned, this finding is consistent with the contention that T2D could be a disease of fiber-type distribution as opposed to mitochondrial function per se.

In addition to the potential influence of obesity and fiber-type distribution that might accompany T2D, it is important to recognise that a reduction in mitochondrial function with T2D is not a universal finding to begin with. For example, Boushel et al. observed a decrease in electron-transport capacity for obese subjects with T2D that was totally attributable to a decrease in mitochondrial content [58]. The authors reasoned that mitochondrial compromise was, therefore, not a cause, but a consequence of T2D [58] or, more specifically, the low level of physical activity that subjects with T2D typically experience [59–61]. This suggestion is supported by longitudinal studies involving training interventions that improve mitochondrial content in patients with T2D [62, 63]. Similarly, mitochondrial defects might be a consequence of T2D-related energetic alterations; for example, hyperglycemia- and hyperlipidemia-induced production of reactive oxygen species (ROS) [64]. Finally, the degree to which any deficit in lipid-oxidising capacity that might be present for subjects with T2D could create a pathological accumulation of IMCL has also been questioned because healthy mitochondrial capacity greatly outstrips oxidative demand. Consequently, a significant reserve capacity exists such that lipid oxidation by patients with T2D should not be limited during their everyday tasks (e.g., during the resting state and for the types of physical activities that would be typical for these patients) [65].

### Skeletal muscle mitochondrial function and insulin resistance

Ambiguity regarding the underlying meaning of any mitochondrial deficiency that accompanies T2D makes it impossible at present to determine whether such deficiency precipitates the elevated IMCL associated with development of the disease. However, findings from studies of IR individuals that do not yet possess diabetes provide important insight. For example, Petersen et al. found that young, lean FH+ with severe IR demonstrate an ~ 80% greater IMCL content and an ~ 30% lower basal rate of mitochondrial ATP synthesis with similar rate of lipolysis compared to insulin-sensitive control subjects matched for age, height, weight and activity level [66]. In a follow-up study, these researchers reported that a reduced rate of mitochondrial ATP synthesis was also present for these subjects during insulin-stimulated conditions (hyperinsulinemic-euglycemic clamp) with flux only increasing by ~ 5% of the basal rate compared to ~ 90% for control subjects [67]. Finally, in another follow-up study, Morino et al. found that FH+ had an ~ 38% lower mitochondrial density compared to control subjects [68]. Collectively, these observations are consistent with the suggestion that IMCL accumulates to pathological levels and induces IR when muscle mitochondria cannot adequately ‘move’ all of the IMCL that is stored.

With respect to dysfunctional mitochondrial lipid oxidation as the ‘inherent defect’ of IR (and, by extension, T2D), the findings by Petersen and colleagues are compelling because the link was established in subjects who were young and not overweight. Consequently, the association could not be attributed to confounding factors like long-term hypoenergetic activity level, chronic hyperenergetic intake and/or resultant obesity. However, it is important to recognise that these subjects were already demonstrating severe IR due to their genetic predisposition; hence, implications regarding the development of IR from this research is only speculative. For example, it has been shown that insulin influences mitochondrial ATP production by increasing mRNA levels from mitochondrial and nuclear genes that encode mitochondrial proteins [69]. Consequently, the mitochondrial defects that were observed for FH+ might have been a consequence as opposed to cause of the severe IR that was inherent for these individuals. It is, therefore, important to also consider findings from longitudinal studies where mitochondrial lipid-oxidising capacity and IS were the independent and dependent variables, respectively. For example, Short et al. had healthy previously-sedentary subjects aged 22–87 years perform 16 weeks of aerobic training that increased the peak rate of oxygen consumption (V̇O_2peak/max_; i.e., the gold-standard index of oxidative capacity), activity of muscle mitochondrial enzymes and mRNA levels of mitochondrial genes and genes involved in mitochondrial biogenesis to a similar extent for all age groups [70]. However, a ‘chronic’ improvement in IS (i.e., increased IS measured ≥96 h after the final training session) was only observed for the ‘younger’ subjects (i.e., aged 20–39 years), which was surprising because the younger subjects also had the smallest window for improvement (IS decreased ~ 8% per decade in this cohort) [70]. This lack of effect of aerobic training on IS despite similar improvement in mitochondrial function for middle-age/older subjects argues against a mitochondrial deficit in the decline of IS at least that which occurs with aging. Conversely, as for the IR associated with FH+, Østergård et al. had sedentary subjects aged 20–50 years perform 10 weeks of aerobic training and found that V̇O_2peak_, oxidative-enzyme activity and IS improved to a similar extent in FH+ subjects with IR and controls [71]. These findings for genetic- compared to age-related IR support the notion of a mitochondrial deficit for the former and again highlight the complex aetiology of the condition. However, while Østergård et al. did find that IS was correlated with V̇O_2max_ at baseline and after 10 weeks of training and that training-induced improvements in V̇O_2max_ were strongly correlated with increased IS, these correlations were present in the control group only [71]. Conversely, for FH+, no such associations were present and, furthermore, AT-induced changes in IS were not correlated with changes in oxidative-enzyme activity in either group [71]. Finally, in a study designed to assess IR in the presence of pharmacological manipulation of mitochondrial capacity, Lagouge et al. showed that Resveratrol, a naturally-occurring activator of NAD-dependent deacetylase sirtuin-1 and, by extension, peroxisome proliferator-activated receptor γ coactivator (a gene associated with mitochondrial biogenesis; PGC-1 α), increases mitochondrial content while also protecting against diet-induced IR in treated mice [72].

In addition to training and pharmacological studies undertaken to assess the association between improvements in mitochondrial function and IS, a number of longitudinal studies have been designed to test the link between mitochondrial decline and development of IR. In one such study, Fleischman et al. reported an ~ 52% decline in mitochondrial DNA/nuclear DNA that was accompanied by a reduction in IS in healthy volunteers exposed to 30 days of administration of the HIV-infection medication stavudine, which is a nucleoside reverse transcriptase inhibitor that carries with it the side effect of mitochondrial toxicity [73]. However, these alterations were not correlated and IMCL (i.e., the proposed intermediate between reduced mitochondrial capacity and IR) was not increased by the intervention [73]. Conversely, Pospisilik et al. found that IS was compensatorily increased in mice after deletion of muscle- and liver-specific mitochondrial flavoprotein apoptosis-inducing factors which created a deficiency in respiratory-chain function that mimicked that which is present in human IR [74]. Moreover, an FA-raising high-fat diet that induced IR in rats also resulted in a compensatory increase in PGC-1 α and other mitochondrial proteins [75]. Although not a universal finding [76], it has been suggested that it is this fat-induced up-regulation of mitochondrial lipid-oxidising capacity and consequent ‘excessive lipid oxidation’ that begets IR due to increased mitochondrial stress, incomplete fatty-acid breakdown and excessive production of ROS [77–79]. Within this schema, it is the resultant loss of insulin responsiveness that causes the mitochondrial dysfunctionality that is typically observed alongside T2D [69, 80].

### The ‘metabolic inflexibility’ of obesity

Ongoing debate regarding the underyling meaning of the mitochondrial deficiency observed for patients with T2D and FH+ subjects underscores the complex aetiology of the loss of IS that characterises these conditions. However, even greater complexity is present when overweight/obesity is part of the pathophysiology. In a landmark study, Kelley et al. measured glucose and FA uptake across the leg alongside indirect calorimetry and found that in both lean and obese subjects, the rate of FA uptake in the basal state (i.e., at rest after an overnight fast) exceeded FA oxidation [81]. This means that regardless of body composition, net storage of IMCL was taking place. Furthermore, the rate of FA uptake was similar between lean and obese subjects. However, lipid oxidation was less for obese subjects, which meant that the net storage of IMCL was greater compared to their normal-weight counterparts [81]. This observation, which suggests that it is the ability to use IMCL that is responsible for IMCL accumulation for obese subjects, lead Kelley et al. to label an impairment in skeletal muscle fatty-acid utilisation as a ‘primary defect’ predisposing (as opposed to resulting from) obesity [81]. Kelley et al. also found a significant negative correlation between leg respiratory quotient (RQ; i.e., the ratio of the rate of CO_2_ production to V̇O_2,_ which reflects substrate proportionality with values ranging from 0.7 to 1.0 for exclusive lipid and carbohydrate oxidation, respectively) and IS, which supports a link between perturbed metabolism of FA and insulin-resistant glucose metabolism [81].

In addition to a reduction in lipid oxidation in the basal state (i.e., during a condition where lipid use should predominate to protect plasma-glucose concentration), Kelley et al. also found that obese subjects did not effectively suppress lipid oxidation during insulin-stimulated conditions. Specifically, during a hyperinsulinemic euglycemic clamp, leg RQ was virtually unchanged from the fasting state (~ 0.90). This is the hallmark of IR. Conversely, for lean subjects, RQ fluctuated from ~ 0.83 to ~ 0.99 for basal and insulin stimulation, respectively [81]. The authors suggest that the ‘metabolic flexibility’ required to appropriately switch between lipid and carbohydrate fuels represents a critical characteristic of healthy metabolic function that is absent with obesity [81, 82].

Kelley et al. contended that an impaired capacity for lipid oxidation is the primary defect of obesity based on the metabolic inflexibility they observed for obese subjects in the basal state [81]. Consequently, researchers began using the difference in RQ across the extremes established during the hyperinsulinemic euglycemic clamp procedure (ΔRQ_clamp-fast_ or ΔNPRER_clamp-fast_ when non-protein respiratory exchange ratio at the mouth is used for the measurement) to evaluate the functionality of substrate switching in different types of subjects [83–86]. However, there have been challenges to the use of both the ‘floor’ and ‘ceiling’ of this measurement for unveiling the underlying cause of metabolic dysfunction. For example, concerns have been raised regarding the degree to which other factors influence basal lipid oxidation. Specifically, Galgani et al. explain that energy balance, macronutrient composition and the substrate content of plasma can all affect the rate of basal lipid oxidation independent of metabolic functionality [87]. Moreover, it is intuitive that basal energy requirements represent a modest metabolic challenge; hence, the absolute rate of lipid oxidation will be low under basal conditions even when metabolic function is preserved and the relative rate of lipid oxidation is high. Therefore, it is, perhaps, the capacity for lipid oxidation during more metabolically-challenging conditions that will more faithfully reveal a defect that sets T2D progression in motion. A dietary-fat overload provides one such challenge [87].

### Metabolic flexibility in response to diet

As previously mentioned, ingestion of a high-fat diet and consequent elevation of plasma FA results in a compensatory decrease in glucose uptake/oxidation by inducing what might be considered a ‘functional’ IR [27, 28, 42, 88]. Accordingly, the ability to rapidly up-regulate lipid oxidation in response to high-fat feeding would be predicted to protect IS while a sluggish capacity for such adaptation could predispose IR and, ultimately, T2D. Findings from a number of investigations support this contention. For example, Heilbronn et al. compared FH+ (i.e., subjects with a strong predisposition for T2D) to age- and fatness-matched individuals with no family history of T2D (FH-) and found that RER increased to a similar extent after a high-carbohydrate meal for both groups [89]. However, FH+ had an impaired ability to reduce RER following ingestion of a high-fat (76%) meal and this difference was present despite similar IS, fasting lipid oxidation and postprandial plasma FA across groups [89]. Importantly, these researchers also observed between-group differences for activation of PGC-1 α and fatty-acid translocase (FAT/CD36; i.e., a mitochondrial-membrane protein involved in the transport of long-chain FA), which is consistent with a genetic predisposition for the impaired response that they observed [89]. Moreover, Ukropcova et al. exposed FH+ and FH- to chronic iso-energetic high-fat (50%) feeding and found that FH+ had a greater sleep RER after three days [90]. They also found that sleep RER in response to the baseline diet that was maintained prior to the three-day period (35% fat) was negatively correlated with IS, which supports an association between the ability to oxidise lipid and maintenance of insulin responsiveness [90]. Finally, Berk et al. assessed substrate oxidation after eucaloric switches from low- (30%) to high- (50%) and high- to low-fat diets and found that Caucasian women demonstrated an increased rate of lipid oxidation, decreased rate of carbohydrate oxidation and decreased RER seven days after increasing fat intake [91]. Conversely, substrate oxidation for African-American women (i.e., a group predisposed to IR and T2D [92]) after the high-fat switch remained fixed. Consequently, African-American women demonstrated a lower rate of lipid oxidation after chronic high-fat intake (181 ± 12 and 231 ± 12 μmol·min^− 1^ for African-American and Caucasian women, respectively) while Caucasian women also achieved a higher rate of carbohydrate oxidation after switching from high- to low-fat intake [91]. Collectively, these findings are consistent with a difference in metabolic flexibility between these two groups and, importantly, these differences remained significant after adjusting for age, waist-to-hip ratio, BMI, percent body fat, fat-free mass, fat mass and adipose-tissue areas (i.e., visceral and subcutaneous deposits). These observations underscore the genetic root of this impairment [89] and this metabolic inflexibility in response to diet resonates when you consider the high-fat content of the standard American diet and the degree to which such a diet likely contributes to the high incidence of obesity [93].

### The metabolic inflexibility of type 2 diabetes

**Fig. 2:**
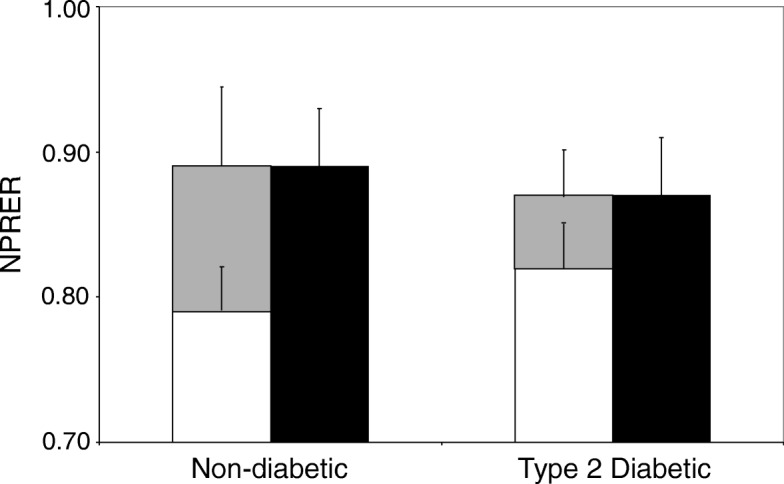
The metabolic inflexibility of type 2 diabetes. Metabolic flexibility is often quantified as the change in non-protein respiratory exchange ratio between metabolic extremes; for example, the fasting state and the metabolic milieu created by supraphysiological insulin stimulation during a hyperinsulinemic euglycemic clamp. In their study, van de Weijer et al. confirmed that subjects with type 2 diabetes demonstrate a higher NPRER (less lipid use) during fasting (open bars) and a lower NPRER (less carbohydrate use) during maximal insulin stimulation (closed bars) [83]. Consequently, these patients demonstrate metabolic inflexibility (reduced ΔNPRER_clamp-fast_; grey bars) compared to their non-diseased counterparts

Given the reduction in metabolic flexibility that FH+ and T2D-predisposed subjects experience in response to dietary manipulations [89–91], it is not surprising that patients with T2D also demonstrate dysfunctional substrate switching. For example, van de Weijer et al. found that RER was higher in the basal state and lower during the insulin-stimulated condition for subjects with T2D compared to age- and BMI-matched controls [85]. This confirmed that the subjects with T2D in their study were metabolically inflexible (see Fig. 2). They also used ^31^P magnetic resonance spectroscopy to show that the phosphocreatine recovery half-time (PCr t_1/2_) following five minutes of knee-extensor exercise was ~ 12.5% slower for the patients with T2D [85]. According to the model of respiratory control proposed by Meyer, PCr kinetics serves as a proxy for V̇O_2_ kinetics and, therefore, provides a measure of oxidative capacity such that a slower response is consistent with impaired mitochondrial function in vivo [94]. Interestingly, van de Weijer et al. also found that PCr t_1/2_ was the only significant predictor of basal RER while whole-body glucose-disposal rate was the only predictor of RER during the insulin-stimulated condition [85]. The latter is consistent with the observation that in patients with T2D, the metabolic inflexibility to glucose during insulin stimulation is exclusively attributable to a decreased rate of glucose availability consequent to an ‘upstream’ disposal rate limitation and, possibly, an inability of insulin to effectively suppress plasma FA [95]. Moreover, these findings help to explain why weight loss improves insulin-stimulated aspects of metabolic flexibility in obese subjects while what appears to be a deeper-seated lipid-related impairment remains unaltered [95]. Indeed, the lowered ceiling that reduces ΔNPRER_clamp-fast_ (i.e., the decreased NPRER_clamp_) appears to be a consequence as opposed to cause of T2D while the inadequate up-regulation of fasting lipid oxidation that elevates the floor (i.e., increases NPRER_fast_) is present in predisposed subjects regardless of development of the disease [95]. Reports of an association between the capacity to oxidise lipid in the basal state and IR for both FH+ [96] and a pooled group of insulin-sensitive and insulin-resistant subjects without T2D [97] also support this contention. Collectively, these findings suggest that NPRER_fast_ alone might be a more important measurement to consider. However, as previously mentioned, using NPRER_fast_ to determine the degree to which the capacity to oxidise lipid is impaired has also been questioned [87].

### Lipid oxidation during exercise

**Fig. 3:**
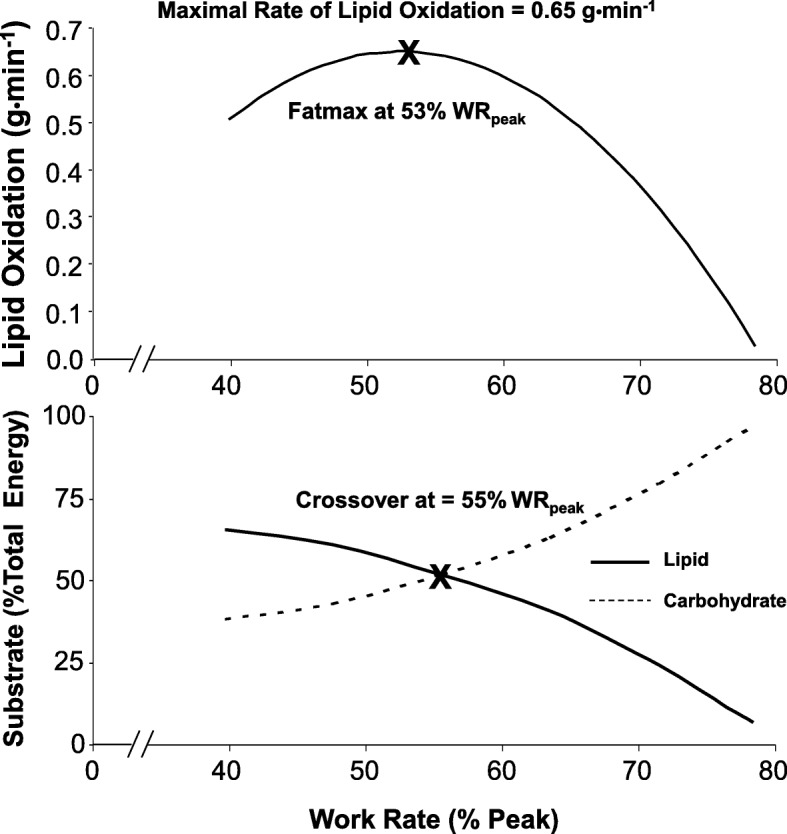
The reciprocal relationship that exists between lipid and carbohydrate use during exercise of increasing intensity. Top panel: As exercise work rate is increased during incremental exercise, the rate of lipid oxidation also increases to reach its maximum value at a mid-range intensity before decreasing and becoming negligible as the highest achievable work rates are encountered. Bottom Panel: The combination of increasing energy expenditure, increasing carbohydrate contribution and the reduced reliance on lipid (see above) as exercise intensity increases results in a ‘crossover’ point beyond which lipid is no longer the predominant fuel. WR_peak_, peak work rate during incremental exercise

In addition to reporting an inverse association between sleep RQ and IS in a group of FH+ and FH- subjects (see above), Ukropcova et al. found that sleep RQ was inversely related to mitochondrial DNA content [90]. However, both groups had a similar decrease in 24-h RQ following the carbohydrate-to-lipid dietary switch and no association between 24-h RQ and mitochondrial DNA content was observed [90]. This means that if a mitochondrial defect was responsible for the metabolic inflexibility demonstrated by FH+, it was only identifiable during sleep, which is difficult to reconcile given the benign metabolic challenge that is present under those circumstances [87]. Conversely, exercise presents a significant challenge to metabolic function that might make it a more appropriate test for functional substrate selectivity. Specifically, it is well established that under normal circumstances, lipid and carbohydrate provide the predominant fuels during aerobic exercise with the relative contribution of each varying markedly depending upon substrate availability and the intensity and duration of the exercise challenge at hand. With regard to intensity, Romijn et al. used stable isotope tracers and indirect calorimetry to evaluate how endogenous fat and glucose metabolism during exercise change with increasing intensity (25, 65 and 85% V̇O_2max_) and found that for five trained cyclists, plasma glucose uptake and muscle glycogen oxidation increased with exercise intensity [98]. This intensity-dependent increase in carbohydrate use contrasted reliance on peripheral lipolysis, which was highest at the lowest intensity with FA release into plasma decreasing at the more intense work rates. However, IMTG lipolysis was stimulated only at the higher intensities. The net effect was that the overall rate of lipid utilisation increases with increasing intensity up to a point after which it decreases and ultimately becomes negligible at the highest work rates (see Fig. 3 Top Panel) [98, 99]. The rate of carbohydrate oxidation increases with increasing intensity in a similar manner; however, in this case, the increase continues such that the relative contribution of carbohydrate outstrips that of lipid (i.e., a ‘crossover’ occurs) once high-intensity efforts are encountered [98, 99] (see Fig. 3 Bottom Panel). In accordance with this reciprocity, the ability to oxidise lipid at a high rate during exercise and, therefore, delay the switch from lipid to carbohydrate predominance might be the functionally-significant capacity that an early-intervention program should be designed to detect and improve.

### The maximal capacity for lipid oxidation and insulin sensitivity

To quantify the capacity to up-regulate lipid oxidation during exercise, the intensities aligned with the maximal rate of lipid oxidation (‘fatmax’) and the crossover point to predominant carbohydrate oxidation during incremental exercise have recently become variables of interest. For example, in 2002, Achten and colleagues assessed 18 moderately-trained cyclists and found that fatmax occurred at ~ 65% V̇O_2max_ with lipid-oxidation rates within 10% of this peak occurring from ~ 55–72% [100]. Conversely, lipid use became negligible above ~ 89% V̇O_2max_ [98]. In a follow-up study, Venables et al. assessed 300 healthy subjects and found that fatmax occurred at 48.3 ± 0.9% of V̇O_2max_ [101]. This established a standard that could prove useful for identifying metabolically at-risk individuals. For example, Robinson et al. assessed 53 young, healthy, recreationally-active men (fatmax at 58 ± 17% V̇O_2max_; range, 21–83%) and found that the maximal lipid oxidation rate (expressed in grams per minute) was positively correlated with IS while V̇O_2max_ and IS shared no such association [102]. Furthermore, in a longitudinal analysis, Venables et al. showed that after four weeks of aerobic training, eight sedentary obese, but otherwise healthy individuals experienced an ~ 44% increase in lipid-oxidation rate during the 30-min exercise bout that was positively associated with the improvement in IS that was also induced by training [103]. Collectively, these studies support the contention that the maximal capacity for lipid oxidation is linked to the IS status of a subject.

### The maximal capacity for lipid oxidation and type 2 diabetes

If the development of IR is associated with a reduction in the maximal capacity for lipid oxidation during exercise, it would stand to reason that subjects with T2D would demonstrate a markedly reduced fatmax. Interestingly, a number of studies have been conducted to confirm this hypothesis, but results have been mixed. For example, Ghanassia et al. compared sedentary subjects with T2D to age-, sex- and BMI-matched sedentary controls with normal glucose tolerance and found that both the fatmax and crossover point occurred at a lower percentage in the patients with T2D (25 ± 1 v. 37 ± 2% and 24 ± 2 v. 39 ± 2% of the peak work rate, respectively) [102]. Indeed, patients with T2D demonstrated a lower lipid oxidation rate per fat-free mass at all five relative exercise intensities that were assessed in that study (i.e., 20, 30, 40, 50 and 60% of the peak work rate) [104]. Another indication of an impaired capacity for lipid oxidation for patients with T2D comes from Suk et al., who compared women with T2D with age-, sex- and BMI-matched controls and found that T2D was associated with a lower fatmax (~ 34 v. ~ 52% of V̇O_2max_) and lipid oxidation rate at fatmax (~ 0.38 v. ~ 0.55 g·min^− 1^) [105]. Specifically, the rate of lipid oxidation was lower at three of the five relative intensities that were investigated (40, 50 and 60 of V̇O_2max_, but not 20 or 30%) [105]. Conversely, Mogensen et al. compared patients with T2D to age-, BMI- and PA-matched FH- controls with normal glucose tolerance and found that both the maximal rate of lipid oxidation (~ 0.3 g·min^− 1^) and fatmax (~ 40% V̇O_2max_) were not different between groups [106]. Furthermore, while fatmax was positively correlated with V̇O_2max_, it did not show any association with insulin-stimulated glucose disappearance rate (R_d_) [106]. In addition, following 10 weeks of aerobic training, fatmax increased to a similar extent in T2D and control group (~ 50% and ~ 40% of V̇O_2max_, respectively) with the change not correlated with the increase in R_d_ that also occurred due to training [106]. Given the importance of determining whether the maximal rate of lipid oxidation and fatmax are, indeed, reduced with T2D, future research should be geared toward clarifying this issue with potential confounding factors like acute and chronic diet, excess body fatness and the insulinemic status of the subject taken into account.

### Lipid oxidation for subjects with type 2 diabetes during moderate-intensity exercise

In addition to incremental exercise with multiple stages to determine the maximal rate of lipid oxidation and fatmax, fuel use for patients with T2D has also been assessed during constant-work-rate (CWR) moderate-intensity exercise and contrasting findings have been reported. For example, in 1995, Martin et al. found that during 40 min of cycling at 60% V̇O_2max_, the hepatic glucose response was normal (production of ~ 2.4 mmol·min^− 1^), but arterial glucose had declined (~ 10%) for non-obese male subjects with T2D compared to subjects without the disease who experienced an ~ 21% rise [107]. This greater carbohydrate utilisation was manifest in a higher RER that was observed during exercise for the patients with T2D [107]. Five years later, Blaak et al. used isotope-infusion techniques during 60 min of cycling at 50% V̇O_2max_ to provide further insight. Specifically, they found a decreased use of plasma-derived FA during exercise for obese subjects with T2D compared to age-, weight- and V̇O_2max_-matched controls [50]. However, subjects with T2D demonstrated an increased reliance on IMTG and/or VLDL such that the whole-body total rate of lipid oxidation during exercise was similar between groups [50]. Employing the same methodology, Mensink et al. confirmed similar fat oxidation during exercise for normoglycemic obese subjects with impaired glucose tolerance (i.e., a group at high risk for developing T2D) due to a ‘trend’ (*P* = 0.07) for a reduced capacity for uptake/oxidation of plasma FA along with an increased reliance on IMTG- and/or VLDL-derived lipid [108]. However, in a follow-up study, subjects with T2D who were not obese (BMI, 28.7 ± 4.2 kg·m^− 2^) demonstrated similar rates of total lipid and carbohydrate utilisation during 60 min of cycling at 40% V̇O_2max_ compared to BMI-matched controls [109]. Moreover, no differences were present in uptake/oxidation of plasma or IMTG-/VLDL-derived lipid energy although the source of carbohydrate energy was altered by the disease (lower oxidation of muscle glycogen and higher plasma glucose use for subjects with T2D) [109]. This suggests that obesity might exert an influence on exercise fuel utilisation independent of T2D status [109]. Finally, Boon et al. also used isotope-infusion methods for a comparison between ‘long-standing’ obese male patients with T2D and normoglycemic controls matched for age, sex, body weight and aerobic capacity during cycling at 50% WR_peak_. These researchers observed no between-group differences for total lipid oxidation (~ 40%), IMTG- and/or VLDL-derived contribution (~ 10%), total carbohydrate oxidation (~ 60%) or plasma-glucose/muscle-glycogen contribution (~ 15 and ~ 45%, respectively) [110]. The reason(s) for this discrepant finding is unclear, but might have to do with the duration for which T2D had been present for the tested subjects. Specifically, in long-standing patients with T2D, characteristics of lipid availability/use secondary to the compensatory hyperinsulinemia that accompanies IR during earlier manifestation of the disease might no longer be present [110]. It is also important to note that the exercise intensities that were assessed in those studies were different (i.e., 40–60% V̇O_2max_), which might also explain equivocal findings (see section entitled ‘Normalisation of Exercise Intensity When Assessing Lipid-oxidising Capacity’ below).

### Lipid oxidation during exercise for obese subjects

As previously mentioned, ~ 85% of patients with T2D are also obese; hence, it is not surprising that studies designed to identify the influence of T2D on exercise lipid use have often been conducted using obese individuals as subjects. Unfortunately, this makes it difficult to tease out the independent influence of T2D. Similarly, as a result of the high prevalence of IR in obese individuals, investigations designed to determine how obesity influences exercise substrate use have typically involved comparisons between groups with differing degrees of IR. Consequently, the complex influence of IR on exercise fuel use (see below) might also be a confounding variable in these studies. With this limitation in mind, the preponderance of evidence suggests that obese individuals do not differ in their capacity for exercise lipid use compared to non-obese control subjects [19, 111–120] although there are reports of a reduced [121, 122] or even enhanced [123, 124] capacity associated with the obese state. Much like was the case with T2D subjects, the distinction appears to be related to the net effect of potential alterations in the ability to uptake/oxidise plasma FA compared to IMTG- and/or VLDL-derived (i.e., non-plasma) sources. For example, Horowitz et al. compared lean and abdominally-obese women matched for V̇O_2max_ during 90 min of moderate-intensity cycling (~ 54% V̇O_2max_) and found that the obese subjects achieved an ~ 25% greater whole-body rate of lipid oxidation despite the fact that whole-body lipolytic activity and plasma FA oxidation were similar between groups [123]. This means that the capacity to derive energy from non-plasma sources (presumably predominantly IMTG) was greater for the obese subjects [123]. Conversely, Goodpaster et al. had obese and lean sedentary males matched for V̇O_2max_ perform 60 min of cycling (50% V̇O_2max_) and found that the percentage of plasma FA oxidation was lower for the obese subjects (51 ± 5 v. 75 ± 10%) [124]. However, despite this impairment, obese subjects once again derived a greater proportion of energy from lipid oxidation (43 ± 5 v. 31 ± 2%). An ~ 50% greater reliance on non-plasma sources was responsible for offsetting and, indeed, reversing the deficit in this case [124]. Finally, Mittendorfer et al. compared lean, overweight and obese subjects during 90 min of cycling (50% V̇O_2peak_) and also found that the relative contribution of plasma FA was decreased while the relative contribution of non-plasma sources was increased in the obese subjects [115]. However, in this case, the changes were relatively balanced such that the contribution of lipid oxidation to total energy transfer (~ 30%) did not differ between groups [115].

### The complex influence of insulin resistance on exercise fuel use

The potential for IR as a confounding factor in the aforementioned research is interesting to consider. As previously mentioned, Robinson et al. found that the maximal rate of lipid oxidation was positively correlated with IS in young, healthy, recreationally-active men [102]. This is consistent with the notion that an impaired capacity for exercise lipid oxidation is associated with the insulin-resistant state. However, this is not a universal finding. For example, Goedecke et al. assessed competitive cyclists and found that despite a wide variation in IS that was present across the group, no correlation between IS and RER at a variety of exercise intensities was found [125]. Moreover, despite the fact that both the peak rate of lipid oxidation and fatmax were higher in overweight subjects with low (0.76 ± 0.02) compared to high (0.89 ± 0.02) resting RER, Rosenkilde found that the difference in IS between groups designated according to that criterion only reached ‘trend’ status (*P* = 0.09) [126]. Finally, Braun et al. examined exercise fuel use for overweight or obese women divided into two groups based on composite insulin-sensitivity index (IS, 7.7 ± 0.9, IR, 3.0 ± 0.7) during 50 min of cycling (45% V̇O_2peak_) and found that RER during the latter stages of exercise was lower in the IR group [127]. The authors suggest that this counterintuitive finding reflects the fact that despite the difference in IR that was present between the two groups, by design, all subjects were normoglycemic. Consequently, exercise fuel use was not confounded by IR-related hyperglycemia and an enhanced capacity for glucose movement from blood to tissues via mass action. In this regard, they suggest that the concomitant reduction in muscle glycogen use which they observed for the IR subjects during exercise represented a ‘downstream’ consequence of initial glycogen levels that were compromised due to the IR [127]. Unfortunately, they did not measure specific lipid sources during exercise and, therefore, could not confirm that the decrease in glycogen use and increase in lipid oxidation associated with IR was a function of a greater reliance on IMCL as would be expected [50, 124]. Regardless of this distinction, however, these findings and those mentioned previously reflect the highly complex nature of IR and suggest that differing degrees of IR and associated hyperinsulinemia and/or hyperglycemia might explain the lack of consensus in the literature regarding the influence of T2D and/or obesity on the capacity for lipid oxidation during exercise.

### Normalisation of exercise intensity when assessing lipid-oxidising capacity

In addition to potential confounding influences of IR and/or obesity, ambiguity regarding exercise fuel use for T2D compared to healthy subjects during CWR exercise could relate to the relative intensity of the exercise challenge presented to the subjects. In the aforementioned study, Venables et al. observed considerable inter-subject variability with fatmax occurring anywhere between 21 and 77% of V̇O_2max_ in their cohort of healthy individuals [101]. Consequently, a methodology that calls for subjects being compared at the same percentage of V̇O_2max_ is inherently flawed because it virtually ensures that some subjects will be working in proximity to their maximal rate of lipid oxidation while others will be exercising far from it. This has resonance because it is now well established that the metabolic and gas-exchange responses to exercise exhibit non-linear characteristics such that a group of subjects working at the same percentage of V̇O_2max_ will encounter considerable inter-subject variability with respect to the physiological challenge at hand [128–130]. Conversely, assigning exercise intensity relative to the point at which the cellular phosphorylation and redox potentials that drive oxidative metabolism initially become altered during incremental exercise (i.e., the lactate or gas-exchange threshold depending upon whether the determination is made from the blood-lactate or gas-exchange response; LT or GET, respectively) provides a way to ensure a more consistent physiological response across subjects [129, 130]. With respect to exercise fuel use, this is particularly relevant because LT/GET also represents the point at which a disproportionate increase in carbohydrate compared to lipid use would be expected to occur. For example, Achten and Jeukendrup found that LT and fatmax were correlated and, indeed, did not differ in a group of endurance athletes [131]. While such coincidence might not be the case in ‘normal’ healthy subjects where fatmax appears to occur at a lower work rate relative to LT (e.g., at ~ 75% LT; i.e., 48 ± 1 vs. 65 ± 1% V̇O_2max_) [101, 132], in lieu of direct measurement of fatmax, it seems reasonable to suggest that exercise work rate should be prescribed relative to LT/GET if the goal is to establish an unbiased comparison of the capacity for lipid oxidation for patients with T2D, IR subjects and subjects who are overweight or obese.

### Aerobic training to improve lipid-oxidising capacity and insulin sensitivity

It is well established by both cross-sectional [133, 134] and longitudinal [135, 136] research that a chronic adaptation to aerobic training is an enhanced capacity for lipid oxidation at a given rate of work. Furthermore, the athlete’s paradox implies that aerobic training that ‘turns over’ IMCL on a regular basis allows elevated levels to be maintained without pathological repercussion. Collectively, these observations suggest that regardless of the cause/consequence relationship shared by elevated IMCL and IR, aerobic training could provide an effective intervention for improving long-term prognosis. However, longitudinal (i.e., ‘training’) studies designed to test this hypothesis have returned mixed findings. Importantly, it has long been known that IS is improved following a single aerobic exercise bout [137–141] with the time course for dissipation of this acute effect believed to be 38–72 h [140, 142]. Consequently, to test for a chronic improvement in IR due to aerobic training, it has been suggested that post-exercise measurements should be performed at least 72 h following completion of the final exercise bout [15]. However, it is intuitive that the ‘chronic’ effect of exercise training on IS will also dissipate with time; hence, testing after a period of inactivity that allows for amelioration of the acute effect might not allow for accurate quantification of exercise’s ‘long-term’ benefit. Moreover, there is growing appreciation for the clinical importance of changes in physiology that occur between single bouts of exercise (i.e., the ‘subacute’ effect) [143]. Hence, with respect to ‘precision medicine,’ transient improvement in IS secondary to the residual effect of daily or near-daily exercise might be an important effect to consider.

Quantification of the chronic effect of aerobic training on IS is also complicated by the fact that the effect appears indirect because it depends on the reduction in body fat that often accompanies the training intervention [15]. For example, Segal et al. had lean and obese men with or without T2D perform four hours of cycle aerobic training per week for 12 weeks with body composition and weight maintained by re-feeding the energy expended during each session and found that despite an ~ 27% increase in V̇O_2max_, IS was not altered in any group [144]. Similarly, Ross et al. compared a group of obese men performing aerobic training (walking/jogging that used 700 kcal per session each day for 12 weeks) with weight loss allowed to a group performing the same aerobic training without weight loss and found an ~ 16% increase in V̇O_2peak_ for both groups [145]. However, IS was only improved for the group that also lost weight and, indeed, the magnitude of this improvement (~ 60%) did not differ compared to that which was achieved in a third group that experienced the same amount of weight loss due to dietary restriction with no exercise training [145]. While this might be interpreted as evidence that similar metabolic benefit can be gleaned from weight loss regardless of whether it is precipitated by a dietary- or exercise-induced negative energy balance, it is important to note that despite similar reductions of body weight in both weight-loss groups (~ 8% of initial body mass), the aerobic-training weight-loss group experienced a greater loss of body fat (~ 1.3 kg), which might provide long-term benefit [145].

Further evidence that weight loss is the key that drives a chronic reduction in IR comes from Schenk et al. who confirmed similar IS improvement alongside an ~ 12% loss of body fat in abdominally-obese women regardless of whether the loss was induced either exclusively by dietary restriction (~ 500–800 kcal/day) or via the same dietary protocol along with aerobic training (45 min of cycling at 85% of the maximum heart rate four days per week) [146]. Interestingly, in addition to an ~ 25% increase in V̇O_2peak_ that differentiated trained from diet-only groups in this study, the similar effect on IS was present even though the trained subjects experienced an ~ 20% increase in resting whole-body fat oxidation [146]. Finally, we recently exposed overweight/obese African American women to a regimen involving a 24-min high-intensity interval-training (HIIT) bout on a cycle ergometer performed three times per week with rigorous control of energy balance to ensure weight stability throughout the 14-week intervention and confirmed no increase in IS due to the exercise training [147]. This has resonance because both GET and fat oxidation at a given submaximal rate of work were improved by the intervention [147].

In addition to improving V̇O_2peak/max_ [144–146] and lipid-oxidising capacity in the resting state [146] and during exercise [147], there is evidence to suggest that weight-stable aerobic training that does not increase IS can favorably alter the composition of lipid deposition. For example, in the study detailed above, Ross et al. showed that aerobic training without weight loss resulted in a reduction in total abdominal fat although not to the same extent as the diet-only and diet-plus-exercise weight-loss groups [145]. Furthermore, Devries et al. had obese and lean sedentary women perform 12 weeks of aerobic training that increased V̇O_2peak_ and lipid-oxidising capacity in both groups and found that while aerobic training did not alter total IMCL content, it did change the characteristics of IMCL accumulation [19]. Specifically, the percentage of IMCL in direct contact with mitochondria was greater after training because stores were redistributed from subsarcolemmal to intermyofibrillar mitochondrial region [19]. This relocation might be responsible for non-pathological compared to pathological IMCL deposition (i.e., the athlete’s paradox; elevated lipid stores that are ‘turned over’ on a regular basis compared to elevated stores that are not). However, aerobic training did not precipitate weight loss in that study; hence, consistent with the aforementioned observations, IS was not improved even in the obese subjects who demonstrated greater IR and fasting plasma insulin in the pre-training measurements [19]. Finally, in a subsequent study, Ross and colleagues found that 14 weeks of daily weight-stable aerobic training (500 kcal expended per session) reduced total (~ 7%), abdominal (~ 10%) and visceral (~ 18%) adipose deposition with no change in IS [148]. Importantly, these improvements were similar to those observed in a diet-induced weight-loss group that lost ~ 6.5% of body weight. Furthermore, when equivalent weight loss was precipitated by AT, the improvements in IS (~ 32%) and total (~ 18%) and abdominal (~ 20%) fat were greater compared to those induced by the diet-only intervention [148]. The authors concluded that regardless of the effect on IS, aerobic training without weight loss provides a useful strategy for reducing total and abdominal obesity, preserving skeletal muscle mass and improving cardiorespiratory fitness [148].

The ability of weight-stable aerobic training to improve numerous aspects of metabolic function without altering IS might seem counterintuitive; however, additional findings from Schenk et al. help to clarify the dissociation. Specifically, unlike resting whole-body lipid oxidation which only increased in the training group in that study (see above), FA mobilisation and uptake increased to a similar extent in both groups [146]. This implies that it is reductions in systemic FA mobilisation/uptake that play the primary role in the increased IS that is observed following a weight-loss intervention [146]. These researchers provided further proof by infusing lipid after the weight loss had occurred to artificially increase FA mobilisation to pre-loss levels to show that the IS improvement was almost completely reversed in both groups despite the augmented capacity for lipid oxidation in the resting state for the trained subjects [146].

The inability for aerobic training to universally improve IS is also apparent when age-related declines in IS are considered. For example, Clevinger et al. determined IS in four groups of subjects divided according to age and trained status (young or older; sedentary or endurance-trained) and found that the normal age-related decline in IS that was present for sedentary subjects (~ 53%) was also present for trained individuals [149]. Specifically, despite the fact that trained subjects demonstrated higher IS in both age groups, an ~ 36% decline was observed for older (60 ± 1 yr) compared to younger (29 ± 1 yr) runners [149]. Moreover, in a subgroup analysis comprising subjects with similar whole-body adiposity, an age-related decline in IS (~ 33%) was only present for the endurance-trained subjects [149]. This further supports the multi-factorial nature of IR and, specifically, how it can be rooted differently in disparate subject populations.

### Type 2 diabetes and excess body-fat deposition

The critical role of weight loss for conveying the improvements in IR that occur with chronic aerobic training underscores the potent effect of excess body-fat deposition on insulin’s regulatory capacity. However, as previously stated, while most subjects with T2D are obese, most obese subjects do not possess T2D despite alterations in insulin action and blood-glucose control. Furthermore, there is growing research interest in a subset of obese individuals without T2D who demonstrate normal IS. However, while the tendency for these ‘metabolically-healthy obese’ individuals to avert the decline in insulin action associated with excess body-fat deposition is intriguing, it is, perhaps, their ability to maintain normal IS in the face of the chronic energetic surplus required to achieve the obese state that is the most remarkable aspect of this phenotype [42, 43, 50].

At present, criteria used to define the metabolically-healthy obese state have not been standardised [6] and controversy exists regarding the degree to which their ‘healthy’ status might simply reflect a transient condition; for example, the slower as opposed to absence of obesity-related progression of metabolic decline. Consequently, findings from prospective cohort studies that detail longitudinal outcomes for metabolically-healthy obese individuals are useful to consider. In the North West Adelaide Health Study, Appleton et al. found that ~ 33% of subjects designated as metabolically-healthy obese at baseline developed metabolic risk and incident T2D during 5.5–10.3 years of follow-up while ~ 67% maintained metabolic health and, accordingly, experienced no additional risk for T2D compared to normal-weight healthy controls [151]. Importantly, for the latter group, in addition to age (≤40 years), the ability to elude the metabolic consequences of obesity was correlated with lower waist circumference, which resonates because central obesity is associated with the low-grade inflammatory response that is linked to IR and associated metabolic aberrations (i.e., the ‘metabolic syndrome’) [152]. For example, Klöting et al. compared morbidly-obese (BMI ≥35 kg·m^− 2^) IS subjects with age-, sex-, fat- and BMI-matched IR individuals and found that increased visceral fat area, increased macrophage infiltration into omental adipose tissue, enlarged adipocyte size in both omental and subcutaneous fat depots and omental adipocyte IR are all associated with IR obesity [153]. These characteristics were further related to dysregulation of circulating adipokines and cytokines including decreased adiponectin and increased progranulin, chemerin, retinol-binding protein-4 and fetuin-A serum concentrations [153]. Specifically, macrophage infiltration and serum adiponectin accounted for 98% of the variation in glucose infusion rate (GIR) during the hyperinsulinemic euglycemic clamp that was used to classify the subjects into IS/IR groups [153]. They also found that estimated hepatic steatosis (the so-called “non-alcoholic fatty liver disease”) was similar to visceral adipose tissue for predicting the variance in IR, which supports the well-established link between fat deposition in the liver and loss of IS [154]. Indeed, there is evidence to suggest that it is intra-hepatic as opposed to visceral adipose deposition that is ultimately responsible for the degree to which IR accompanies obesity [155].

With respect to lifestyle factors, data from the North West Adelaide Health Study suggest that while the metabolically-healthy obese phenotype was associated with lower levels of physical activity compared to normal-weight healthy subjects, these individuals were more likely to engage in moderate-to-high-level physical activity compared to their metabolically at-risk obese counterparts [151]. This is consistent with evidence from both cross-sectional and longitudinal studies which confirms that higher cardiorespiratory fitness is a characteristic of the phenotype [156]. However, neither baseline nor follow-up level of moderate-to-high physical activity was associated with the ability for obese subjects to maintain the metabolically-healthy status over time [151]. This is, perhaps, surprising given the degree to which aerobic training can favourably alter fat-deposition pattern even when body weight remains unchanged (see above) [19, 145, 157, 158]. Future research is required to provide insight on how habitual physical activity and/or exercise might contribute to achievement of the metabolically-healthy obese phenotype independent of or in concert with other lifestyle factors (e.g., dietary composition) and genetics.

### Metabolically-obese Normal weight

Similar to metabolically-healthy obese individuals, there is growing research interest in individuals who are of normal weight, but ‘metabolically obese’ because this phenotype provides an opportunity to study the metabolic decline that culminates in T2D independent of the influence of excess body fatness and/or chronic positive energy balance. In one such study, Scott et al. set out to determine the role of common genetic variants shown to be associated with IR in the aetiology of T2D when obesity is not present and confirmed an association for genetically-predicted IR with incident T2D completely independent of BMI [159]. Indeed, this association was present even in normal-weight subjects with the lowest waist circumference (< 94.0 and 78.5 cm for male and female subjects, respectively) [159]. Importantly, these variants are associated with impaired adipose expandability and ectopic fat accumulation, which means that genetic scores for IR implicate an impaired capacity to store lipids in the aetiology of T2D even when obesity and chronic positive energy balance are not part of the equation. However, there do appear to be qualitative differences when the IR-to-T2D pathway is not complicated by the obese state. For example, in a recent review article, Stefan et al. determined the degree to which four major risk phenotypes (non-alcoholic fatty liver disease, visceral adipose tissue, ratio of subcutaneous abdominal fat mass to total fat mass and ratio of subcutaneous gluteofemoral fat mass to total fat mass) might account for the metabolically-unhealthy normal-weight state in 981 subjects at increased risk of cardiometabolic disease based upon body weight, FH+ status, elevated blood glucose level or a history of gestational diabetes in women [11]. In addition to identifying the prevalence of these phenotypes in metabolically-unhealthy normal-weight individuals compared to normal-weight healthy subjects, these authors also sought to discover how their influence might differ compared to that which is present with the overweight/obese unhealthy state [11]. As there is no universal definition of what constitutes the metabolically-healthy state in the literature, in their review, Stefan et al. defined it as one for which fewer than two parameters of the metabolic syndrome were present. These researchers found that compared to metabolically-healthy normal-weight individuals, the most prevalent risk phenotype for a reduction in metabolic health for normal-weight individuals was insulin-secretion failure with these individuals also more frequently demonstrating IR, non-alcoholic fatty liver disease, visceral obesity, a low percentage of subcutaneous gluteofemoral fat mass, low cardiorespiratory fitness and increased carotid intima-media thickness (a measure that indicates the extent of carotid atherosclerotic vascular disease) [11]. Importantly, a number of these characteristics contrasted what was observed for metabolically-unhealthy overweight/obese subjects who demonstrated weaker increases in the prevalence of IR, low percentage of subcutaneous leg fat mass and impaired cardiorespiratory fitness with stronger increases in the prevalence of non-alcoholic fatty liver disease and visceral obesity. These findings regarding differences in body-fat compartmentalisation are particularly intriguing because the optimal pharmaceutical treatment strategy in this case (i.e., drugs that promote adipocyte differentiation to address the impaired capacity for expansion of subcutaneous adipose tissue in the lower body) would be different compared to what might be employed for metabolically-unhealthy obese patients [11]. However, the researchers warn that they only assessed Caucasians in this study, which is important because compared to Caucasians (21.0%), the metabolically-unhealthy normal-weight phenotype is more prevalent in Chinese Americans (32.2%), African Americans (31.1%), Hispanics (38.5%) and South Asians (43.6%) [157]. Moreover, they only included middle-aged subjects. Consequently, findings from this study might not be reflective of what would be present in other ethnic or age groups. Nevertheless, these results confirm that the pathway to T2D can be different for predisposed individuals when excess body-fat storage and chronic positive energy balance are not part of the sequela.

### Exercise as medicine for type 2 diabetes

In 2010, the ACSM and the American Diabetes Association (ADA) issued a joint position statement that included a review of the research concerning how exercise or physical activity might positively affect physical fitness, morbidity and mortality in individuals with T2D [20]. Importantly, the statement delineates acute from chronic training effect by recognising that mild- and moderate-intensity exercise can result in transient improvements in systemic insulin action and, by extension, blood-glucose control from 2–72 h post exercise [20]. The time course of this improvement appears to be related to both the duration and intensity of the preceding exercise bout with age, pre-exercise level of control and state of physical training also playing a role [20]. Furthermore, with respect to this acute effect, a combination of aerobic training and resistance training might be more effective for blood-glucose management in patients with T2D compared to either type of exercise performed exclusively [20]. However, the degree to which positive findings regarding such ‘combination training’ reflect a synergistic effect due to disparate training adaptations (e.g., enhanced peripheral insulin action due to aerobic training plus augmented glucose storage due to resistance training) as opposed to simple volume differences remains to be clarified [20]. Nevertheless, data from two recent systematic reviews and network meta-analyses provide compelling evidence that combining the two types of exercise training is the best strategy for patients with or at risk for T2D. For example, a program that combines aerobic and resistance training is superior for favourably altering body composition in overweight/obese subjects because aerobic training results in a more pronounced reduction of body weight, waist circumference and fat mass while resistance training elicits a greater increase of lean body mass [160]. Furthermore, for patients with T2D, combining the two forms of training brings the greatest reduction in HbA1c, fasting glucose, triaglycerols, high-density lipoproteins, diastolic blood pressure and body weight compared to either type of exercise performed exclusively [161]. However, with respect to these findings, the authors warn that interpretation with regard to clinical relevance is limited by the low-to-moderate quality of the included studies and the amount of information provided on clinically-important outcomes and adverse effects of exercise [161].

With respect to chronic improvements in disease prognosis for patients with T2D, the position statement recognises that exercise training can elicit positive adaptations that result in improved insulin action, blood-glucose control and muscle-lipid characteristics [20]. Specific training-induced adaptations suggested to be beneficial in this regard include enhanced expression and/or activity of proteins involved in glucose metabolism and insulin signaling (e.g., glycogen synthase, GLUT4), improved capacity for lipid oxidation and increased IMCL storage [20]. Once again, the role that resistance training and resultant muscular hypertrophy might play in addition to traditionally-prescribed aerobic training is mentioned [20]. For example, Bweir et al. found that a group of patients with T2D experienced a greater decrease in hemoglobin A1c after 10 weeks of progressive resistance training compared to the decrease due to treadmill training that required the same heart-rate response (60–75% maximum) for age- sex- and disease-matched subjects (an ~ 18 and ~ 8% decrease, respectively) [162]. These findings support the contention that an exercise program designed to chronically improve blood-glucose control for patients with T2D should include a resistant-training component [20].

In addition to explaining modes of exercise that can benefit patients with T2D, the ACSM/ADA position statement includes specific recommendations regarding exercise prescription. For example, for aerobic training, the recommendation is for patients with T2D to perform at least 150 min per week; specifically, three weekly sessions with no more than 48 h interspersed [20]. The objective of this high-frequency paradigm is to ensure that the acute increase in insulin action that follows an aerobic-training session is in effect continuously. It is suggested that aerobic training be of at least moderate intensity (40–60% V̇O_2max_) although additional benefit might be attainable with vigorous efforts (> 60% V̇O_2max_) [20]. For example, a meta-analysis by Boulé et al. suggests that aerobic-training intensity predicts the post-intervention improvement in hemoglobin A1c to a larger extent compared to volume of aerobic training performed [163]. With regard to resistance training, the recommendation is for at least two, but ideally three weekly sessions on non-consecutive days using either moderate or heavier loads (i.e., 50% or 75–80% of the one-repetition maximum weight, respectively) with the latter considered optimal for improvements in strength and insulin action [20]. The resistance-training protocol should include at least 5–10 exercises involving the major muscle groups with sets continued to near failure (e.g., starting at 10–15 repetitions and progressing over time to 8–10) [20]. It is recommended that one set will suffice, but as many as four might be optimal for strength gain [20]. Finally, patients with T2D are advised to increase their total daily unstructured physical activity to assist in the control of body weight [20]. This is important because it is well established that individuals with T2D engage in less physical activity; for example, a survey of the US population in 2003 revealed that only 39% of adults with diabetes were physically active compared to 58% of healthy counterparts [59]. Moreover, recent accelerometer data confirms lower total activity counts for older individuals (> 60 years) with T2D compared to older subjects with normal blood-glucose levels [60]. Whether this reduction is a cause or consequence of T2D cannot be ascertained from this cross-sectional study; however, it is interesting to note that subjects diagnosed with prediabetes demonstrated similar total activity counts compared to healthy controls [60]. It has also been shown that in addition to a reduction in the total amount of physical activity, individuals with T2D appear to engage in lower-intensity efforts compared to healthy subjects [61]. Collectively, these related to physical activity facilitate the positive energy balance and associated metabolic decline that is typically present for patients with T2D [150].

The joint position statement regarding exercise and T2D also provides evidence statements concerning physical activity as an intervention to prevent or delay the onset of T2D in at-risk subjects without the disease. For example, in a population-based prospective study, Wei et al. assessed 8633 individuals without diabetes at least twice and found that during an average follow-up visit after six years, 149 had developed T2D while 593 had developed impaired fasting glucose [164]. After age, cigarette smoking, alcohol consumption and parental diabetes were considered, the least fit 20% of the cohort according to treadmill V̇O_2max_ at baseline had a 1.9-fold risk for impaired fasting glucose and 3.7-fold risk for T2D compared to the most fit 40% of the cohort. Age, BMI, blood pressure, triglyceride level, and FH+ status were also directly related to the risk of developing T2D [164]. Similar results were observed when the Diabetes Prevention Program Research Group conducted a large, randomised clinical trial involving US adults at high-risk for T2D. Specifically, these researchers exposed 1079 subjects that did not possess T2D but did demonstrate elevated plasma glucose during fasting and two hours after a 75-g oral glucose load to a longitudinal lifestyle-intervention program that included 150 min of physical activity per week. The program was designed to facilitate weight loss of ~ 7% and subjects in this group were compared to similar subjects treated with a blood-glucose control medication (metformin) or placebo [165]. Throughout an average follow-up period of 2.8 years, the cumulative incidence of T2D was lower compared to placebo in both intervention groups; however, the reduction was greater with the lifestyle-intervention strategy (58 vs. 31%) [165]. Indeed, lifestyle intervention prevented one case of T2D per seven persons treated for three years and, importantly, this preventative effect was similar in both sexes, for all ethnicities and in older subjects [165]. Unfortunately, given the methodology, teasing out the independent effect of increased physical activity compared to those of dietary changes and weight loss was not possible. However, after reviewing all of the relevant research, authors of the position statement suggest that physical activity seems to play a role in preventing T2D across ethnic groups and in both sexes and provide further clarification by stressing that this applies to moderate-intensity aerobic training while the role of resistance training in preventing T2D has not yet been investigated [20]. They conclude with an evidence statement which indicates that at least 2.5 h/week of moderate to vigorous physical activity should be undertaken as part of lifestyle changes that are appropriate for T2D prevention in high-risk adults [20].

### The key variable for prescribing exercise as precision medicine for insulin resistance

The ACSM/ADA joint position statement provides important evidence-based recommendations that can help to inform the design of exercise-training regimens for patients with T2D and at-risk individuals [20]. However, if exercise is to truly be considered as medicine for treating the underlying cause of T2D, greater detail is necessary. Specifically, consistent with the concept of ‘precision medicine’ [166], it stands to reason that when exercise is *prescribed* as a treatment modality, program variables should be determined precisely in accordance with specific characteristics of the individual. Of particular import in this regard is determination of intensity of effort, which might very well represent the key variable when prescribing exercise as medicine for IR. Specifically, it is conceivable that three distinctly different intensity paradigms could be optimal for treating IR and delaying/preventing its progression to T2D. For example, if a mitochondrial defect predisposes the pathological progression, an exercise intensity that maximises improvements in mitochondrial function would be warranted. There is a growing body of research which suggests that HIIT is specific for this purpose [167, 168]. This approach, which provides a time-efficient way to rapidly improve insulin action for patients with T2D and prediabetes [169], involves periods of high-intensity (HIIT) or all-out (sprint-interval training; SIT) exercise interspersed with periods of rest or low-intensity effort. However, while this type of exercise would maximise peripheral stimulation, the contribution of lipid to exercise fuel use at this intensity would be negligible (Fig. 3). Conversely, if mitochondrial function is sufficient [63] and ectopic fat accumulation is at the root of the pathology, a low-intensity approach that allows the maximal rate of lipid oxidation to be sustained might be preferred to maximally perturb the IMCL pool [101, 170–172] and/or change its relationship with muscle mitochondria [19]. Indeed, such a protocol has been used to improve the ability to oxidise lipid during exercise for patients with T2D [173]. Finally, if weight loss is the ultimate objective of the training, exercise at the maximum sustainable pace (e.g., at a metabolic rate that exceeds that which allows for the maximal rate of lipid oxidation, but is aligned with the work-rate/time asymptote for high-intensity exercise; i.e., the ‘critical power/velocity’) would provide for the duration/intensity interaction that results in the greatest energy use. This approach might also be preferred because a program for obese women with metabolic syndrome that included CWR walk/running at a work rate situated midway between that which is aligned with LT and V̇O_2peak_ (i.e., at 50%∆, which would, on average, be in proximity to the critical velocity [174]) reduced total and subcutaneous abdominal fat and visceral adipose tissue while one that involved walk/running at/below LT with similar energy expenditure (~ 2000 kcal/wk) did not [175]. The reason(s) for this difference despite similar energetic outlay is/are unclear, but might have to do with greater post-exercise energy expenditure and fat oxidation due to an intensity-driven release of lipolytic hormones (e.g., growth hormone and epinephrine) [175].

**Fig. 4:**
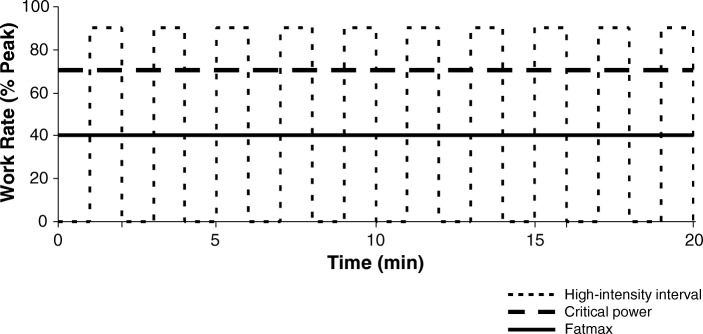
Three distinctly different intensity paradigms that could be optimal for preventing/treating insulin resistance and its progression to type 2 diabetes. Constant-work-rate exercise at the ‘fatmax’ would allow for the highest rate of lipid turnover while constant-work-rate exercise at the ‘critical power’ would result in the greatest sustainable rate of energy use. Even higher intensities can be achieved by employing high-intensity interval training; however, in this case, intermittent periods of rest or low-intensity exercise must be interspersed to allow the exercise bout to be continued. See text for further details regarding advantages and disadvantages of each of these approaches

With respect to determining the intensity of effort that is optimal for delaying/preventing the progression of IR to T2D, it is apparent that arguments can be made for three distinctly different approaches (see Fig. 4). Consequently, more research to compare these training paradigms in different types of at-risk subjects and at different points of disease progression are warranted. However, another possibility is that a program that combines all three types of aerobic training on different days of a training cycle might be best suited for spanning the range of adaptations that could improve prognosis. Interestingly, such a ‘periodised’ approach to training has long been a staple of the programs followed by endurance athletes that might typically involve ‘easy,’ ‘steady,’ ‘tempo’ and high-intensity-interval training over the course of a week [176]. The degree to which such an approach is both effective and safe for individuals with IR/T2D requires future research attention.

## Conclusion

**Fig. 5:**
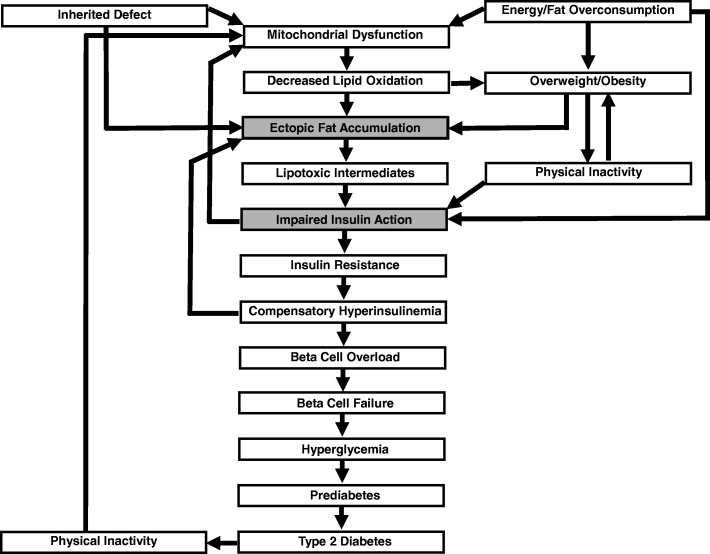
Multi-factorial sequence of events that can potentially explain the progression to T2D. It is well established that genetics and/or an obesogenic lifestyle can precipitate a series of events that culminate in type 2 diabetes. While cause/consequence characteristics continue to be debated, it is undeniable that impaired insulin action is a key aspect of the pathology with ectopic fat accumulation also playing a central role

The cause/consequence characteristics of the relationship between pathological fat deposition and/or mobilsation, elevated and/or poorly-distributed IMCL, compromised mitochondrial structure and/or function, an altered ability to use lipid as fuel and IR remains to be clarified (see Fig. 5). However, the well-established association between IR and the pathological progression to T2D in both obese and normal-weight individuals suggests that a treatment protocol should be geared toward both symptom and cause. With respect to aerobic training and IR, it appears that the independent effect is predominantly acute; for example, a 48-h increase in IS upon completion of an exercise bout. However, research also suggests that such exercise can aid in the achievement of a chronic improvement in IS by facilitating the negative energy balance needed to elicit the requisite body-fat loss. Regular exercise training can also alter lipid-deposition pattern resulting in a more favourable distribution of both adipose and intramuscular stores. However, the training intensity/intensities best suited to precipitate these changes remains to be determined. With numerous issues still unresolved, further research is required before exercise can faithfully be prescribed as ‘precision medicine’ to combat IR and its progression to T2D.

## Abbreviations

50%∆: Work rate situated midway between that aligned with LT and V̇O_2peak/max_
ACSM: American College of Sports Medicine
ADA: American Diabetes Association
AMA: American Medical Association
BMI: Body Mass Index
CWR: Constant Work Rate
FA: Fatty Acids
FAT/CD36: Fatty-acid Translocase
Fatmax: Intensity aligned with the maximal rate of lipid oxidation
FH-: No family history of TD2
FH +: Family history of TD2
GET: Gas Exchange Threshold
GLUT4: Insulin-regulated glucose transporter
HIIT: High-intensity Interval Training
IMCL: Intramyocellular Lipid Content
IMTG: Intramyocellular Triglyceride
IR: Insulin Resistance
IS: Insulin Sensitivity
LT: Lactate Threshold
NPRER: Non-protein Respiratory Exchange Ratio
NPRER_clamp_: NPRER during hyperinsulinemic-euglycemic clamp
NPRER_fast_: NPRER during fasting
NPRQ: Non-protein Respiratory Quotient
NPRQ_clamp_: NPRQ during hyperinsulinemic-euglycemic clamp
NPRQ_fast_: NPRQ during fasting
PCr t_1/2_: Phosphocreatine half-time
PGC-1 α: Peroxisome Proliferator-activated Receptor γ Coactivator
R_d_: Glucose Rate of Disappearance
RER: Respiratory Exchange Ratio
ROS: Reactive Oxygen Species
RQ: Respiratory Quotient
SAT: Subcutaneous Adipose Tissue
SIT: Sprint Interval Training
T2D: Type 2 Diabetes Mellitus
VLDL: Very-low-density Lipoprotein
V̇O_2peak/max_: Peak/maximal Rate of Oxygen Consumption
WR_peak_: Peak Work Rate
ΔNPRER_clamp-fast_: Difference in NPRER_clamp_ v. NPRER_fast_
ΔRQ_clamp-fast_: Difference in RQ_clamp_ v. RQ_fast_
